# Anxiety and depression disorders in patients with pulmonary arterial hypertension and chronic thromboembolic pulmonary hypertension

**DOI:** 10.1186/1465-9921-14-104

**Published:** 2013-10-09

**Authors:** Dominik Harzheim, Hans Klose, Fabiola Peña Pinado, Nicola Ehlken, Christian Nagel, Christine Fischer, Ardeschir Ghofrani, Stephan Rosenkranz, Hans-Jürgen Seyfarth, Michael Halank, Eckhard Mayer, Ekkehard Grünig, Stefan Guth

**Affiliations:** 1 Centre for Pulmonary Hypertension, Thoraxclinic University Hospital Heidelberg, Amalienstrasse 5, Heidelberg D-69126, Germany; 2 Department of Pneumology, University Hospital of Hamburg, Hamburg, Germany; 3 Department of Human Genetics, University of Heidelberg, Heidelberg, Germany; 4 Department of Pneumology, University Gießen-Marburg, Gießen, Germany; 5 Department of Cardiology, University of Köln, Köln, Germany; 6 Department of Pneumology, University of Leipzig, Leipzig, Germany; 7 Department of Pneumology, University of Dresden, Dresden, Germany; 8 Department of Thoracic Surgery, Kerckhoff-Klinik Bad Nauheim, Bad Nauheim, Germany

**Keywords:** Pulmonary hypertension, Mental disorders, Quality of life, Survival

## Abstract

**Background:**

The objective of this prospective study was to assess the prevalence of anxiety and depression disorders and their association with quality of life (QoL), clinical parameters and survival in patients with pulmonary hypertension (PH).

**Methods:**

We prospectively assessed 158 patients invasively diagnosed with pulmonary arterial hypertension (n = 138) and inoperable chronic thromboembolic PH (n = 20) by clinical measures including quality of life (QoL, SF-36 questionnaire), cardiopulmonary exercise testing and six minute walking distance and by questionnaires for depression (PHQ-9) and anxiety (GAD-7). According to the results of the clinical examination and the questionnaires for mental disorders (MD) patients were classified into two groups, 1) with moderate to severe MD (n = 36, 22,8%), and 2) with mild or no MD (n = 122). Patients were followed for a median of 2.7 years. Investigators of QoL, SF-36 were blinded to the clinical data.

**Results:**

At baseline the 2 groups did not differ in their severity of PH or exercise capacity. Patients with moderate to severe MD (group 1) had a significantly lower QoL shown in all subscales of SF-36 (p < 0.002). QoL impairment significantly correlated with the severity of depression (p < 0.001) and anxiety (p < 0.05). During follow-up period 32 patients died and 3 were lost to follow-up. There was no significant difference between groups regarding survival. Only 8% of the patients with MD received psychopharmacological treatment.

**Conclusion:**

Anxiety and depression were frequently diagnosed in our patients and significantly correlated with quality of life, but not with long term survival. Further prospective studies are needed to confirm the results.

## Introduction

Pulmonary Hypertension (PH) is defined as an increase in mean pulmonary arterial pressure (PAP) ≥25 mmHg at rest diagnosed by right heart catheterization [1,2]. At time of diagnosis patients are usually severely affected with impaired exercise capacity and shortness of breath according to WHO functional class II-IV due to elevated pulmonary artery pressure, increased pulmonary vascular resistance and right heart failure [3-5].

In consequence, patients with PH have to manage various life stressors, such as physical burdens, unclear prognosis, high cost of treatment, and often unemployment, which can have a psychological impact and may affect patients’ social contacts and relationships [6,7]. These stressors may lead to the development of mental disorders (MD) as depression and anxiety, which have been detected in 35% of PH-patients [6]. In this study the most common disorders major depression and panic disorder have been related to the degree of symptoms and functional impairment. The prevalence of major depression increased from 7.7% in patients with NYHA functional class (FC) I to 45% in FC IV [6]. The prevalence of frequent panic attacks increased up to 25% in patients NYHA FC IV. Only 24.1% of patients with PH and mental disorders received psychopharmacological or psychotherapeutic treatment [6]. Recent studies confirmed these findings and detected major depression in 25% of PAH patients of the REVEAL registry [8] up to 55% in PAH patients seen in two PH referral centers in the United States [9]. The prevalence of mental disorders in patients with inoperable chronic thromboembolic PH (CTEPH) has been less well assessed.

In patients with other chronic diseases as coronary artery disease or chronic obstructive lung disease, depression was also strongly associated with functional impairment [10] leading to increased mortality [11].

For patients with PH it is unclear if mental disorders as depression and anxiety lead to an impaired quality of live and impaired prognosis and may be addressed in therapy algorithm. Therefore, the primary objective of our study was to examine the prevalence of mental disorders as anxiety and depression in patients with PAH and inoperable CTEPH who have been stable under optimized PH-targeted medication and to analyze its association with exercise capacity, quality of life and survival.

## Methods

### Study population and design

We prospectively included patients with PAH and inoperable CTEPH who have been stable under optimized PH-targeted medical treatment for at least 2 months. Further inclusion criteria were: age between 18 and 80 years and WHO-FC I – IV. The status “inoperable CTEPH” had been confirmed by experienced PEA-surgeons (SG, EM). Patients had to be under optimized medical therapy for PAH (as endothelin-antagonists, inhaled or parenteral prostanoids, phosphodiesterase-5-inhibitors, anticoagulants, diuretics, and supplemental oxygen) for at least 2 months before entering the study. The diagnosis PAH, inoperable CTEPH was established at the participating centers according to current guidelines [2,5]. Patients with severe comorbidities as interstitial lung disease, untreated left heart disease or known mental disorders at the time of diagnosis by right heart catheterization were excluded from the study. All patients underwent a detailed clinical work up including a careful medical history asking for mental disorders, ECG, laboratory testing with Serum N*-*terminal pro brain natriuretic peptide (NTproBNP), 6-minute walking distance under standardized conditions [12], echocardiography at rest and during exercise, lung function tests, cardiopulmonary exercise testing (for exclusion of comorbidities) and right heart catheterization. In case of suspected CTEPH pulmonary angiography was performed. Screening for mental disorders was performed by medical history and using the Patient Health Questionnaire (PHQ-9) and Generalized Health Anxiety Disorder 7-item questionnaire (GAD-7). Patients were then divided into two groups: group 1 with no or only mild MD, group 2 with moderate or severe mental disorder (PHQ-9 ≥ 10 and/or GAD-7 ≥ 10). Analysis of quality of life was performed using the short form health survey 36 (SF-36) questionnaire. The clinicians who performed clinical follow-up assessments and treatment were completely blinded to the results of the MD- and quality of life questionnaires. The investigators who analysed the SF-36 questionnaires were blinded to the results of the PHQ-9- and GAD-7-questionnaire and vice versa. Survival rate has been assessed in 2012 by phone contact or by a control visit. The investigators of the clinical data and survival rate have been blinded to the results of the SF-36-, PHQ-9- and GAD-7-questionnaires. Two independent investigators performed a quality check of the database of all questionnaires. All patients gave written informed consent for this study, which was approved by the Ethics Committee of the University of Heidelberg.

### Assessment of mental disorders using PHQ-9 and GAD-7 questionnaires

Both questionnaires were self-completed by the patient in written form and refer to the symptoms of the patients within the last 2 weeks.

The Patient Health Questionnaire (PHQ-9) was developed in 1999 as a self-reporting questionnaire allowing a criteria-based diagnosis of depression in primary care [13]. PHQ-9 had an excellent reliability and validity for the diagnosis of depression and consists of the nine diagnostic criteria items measuring the severity of depressive symptoms [14]. Spitzer et al. [15] recommended categorizing the PHQ-9 total score into four severity groups: no symptoms of depressive/anxiety disorders (0–4), mild (5–9), moderate (10–14), severe (15–21) symptoms. For our study we divided the groups in 1) none-mild depression using the cut-off score of ≤9. Members group 2) with moderate to severe depression had the score ≥10.

The generalized anxiety disorder questionnaire (GAD-7) is a self-reporting questionnaire to diagnose anxiety using 7 diagnostic items [15]. The diagnostic score of each item of the questionnaire ranges from 0 (not at all) to 3 (nearly every day). To estimate the severity of symptoms the total summation score ranges from 0 to 21. The PHQ-9 is divided into minimal (score 0–4), mild (5–9), moderate (10–14) and severe (15–19) anxiety. The questionnaire has a high reliability and validity for the diagnosis of major depression [16]. For our study we divided the groups in 1) none-mild anxiety using the cut-off score of ≤9. Members of group 2 with moderate to severe anxiety had the score ≥10.

### SF-36 questionnaire

The SF-36 consists of 36 items divided into 8 subscales: physical functioning, role limitations relating to physical health, bodily pain, general health perception, vitality, social functioning, role limitation relating to mental health, and mental health. Each question is rated on an ordinal scale with two to six categories. The score of each dimension is the addition of the item scores of the related dimension further transformed to a score of 0–100, with higher values representing better perceived health-related quality of life [17].

### Cardiopulmonary exercise testing and echocardiography

At baseline, a symptom-limited exercise test was performed during supine bicycle exercise as described previously [18]. The exercise testing began at 25 Watt (W) with a stepwise increment of 25 W every two minutes. Systolic pulmonary artery pressure (PASP), systolic (RRsys) and diastolic (RRdiast) systemic blood pressures, Work load, heart rate, minute ventilation (VE), oxygen uptake (VO_2_), oxygen pulse (VO_2_/heart rate), and oxygen saturation (SaO_2_) were measured continuously. The anaerobic threshold was determined using the V-Slope method [19]. Peak VO_2_ was defined as the highest 30-second average value of oxygen uptake during the last minute of the exercise test. Borg dyspnea index (with 6 representing no exertion and 20 maximal exertion) [20] was inquired immediately after the test. Two-dimensional and Doppler-echocardiographic recordings were performed immediately before and during the cardiopulmonary exercise testing using 2.5 MHz Duplex probes and conventional equipment (Vivid 7, GE Healthcare, Milwaukee, Wisconsin) by experienced cardiac sonographers.

### Follow-up assessment

In June 2012 all participating patients were interviewed either by telephone or at a control visit in the Thoraxclinic Heidelberg using a half-structured questionnaire, including structured questions also leaving space for further clarification and remarks. The patients were asked for present symptoms, current medication, any further cardiac events that might have occurred since last observation. In the situation where the index patient was deceased, date of death was recorded and treating physicians and/or relatives were contacted for the cause and circumstances of death.

### Statistical methods

Statistical analyses were conducted by a statistician (CF), the results were expressed as mean ± standard deviation. Baseline was defined as the day when the patient completed the questionnaires. All clinical and haemodynamic characteristics of patients at baseline were analysed by descriptive statistics. The two subgroups were compared by two-sided Student´s t-test.

For comparison of categorical variables between groups chi-square test was used. In case of larger tables Craddock-Flood test and Haldane-Dawson test were used. The Craddock-Flood Test is recommended for large tables with small degrees of freedom and low-frequency cells, whereas the Haldane-Dawson test is used for contingency tables with more than five rows and/or columns and small sample sizes.

Correlation between the MD anxiety and depression with subscores of the SF-36 were analysed by the robust Kendalls Tau correlation coefficient. For inner-group comparisons non-parametric Kruskal Wallis test was used. All tests were two sided and p-values <0.05 were considered statistically significant. All enrolled patients were included in the survival analysis. Survival was estimated from baseline until June 2012 (the end of follow-up in this study) by Kaplan-Meier analysis. Patients with deaths were counted as endpoints. We analysed whether the two groups with mild to moderate and severe MD differed in their survival.

All analyses were performed using IBM SPSS 20 (SPSS Statistics V20, IBM Corporation, Somers, New York).

## Results

### Study population

We prospectively included 172 consecutive patients diagnosed with PAH or inoperable CTEPH. Ten patients had to be excluded due to comorbidities as severe lung or left heart disease, 4 were excluded due to incomplete PHQ-9 and GAD-7 questionnaires. Thus, the final study group consisted of 158 patients (45 males, 113 females, mean age was 56 ± 16 years: 138 with PAH, 20 with inoperable CTEPH).

Based on the scores of both the PHQ-9- and GAD-7- questionnaires, patients were divided into two subgroups. Group 1 included 122 patients without or with mild mental disorder (39 males and 83 females, mean age, 57 ± 16 years) and group 2 included 36 patients with moderate to severe anxiety and/or depression (6 males and 30 females, mean age, 53 ± 16 years) (Table 1). Thus, within the entire group 22.8% of patients presented with moderate to severe anxiety or depression disorder. There were only few patients (7.0% GAD-7 and 5.7% PHQ-9) with no signs of MD (Table 1).

**Table 1:** Results of GAD-7 and PHQ-9 questionnaires

**Symptoms**	**Anxiety disorder (GAD-7)**	**Depression disorder (PHQ-9)**
No symptoms	11 (7.0%)	9 (5.7%)
Minimal symptoms	75 (47.5%)	62 (39.2%)
Mild symptoms	55 (34.8%)	55 (34.8%)
Moderate symptoms	13 (8.2%)	21 (13.3%)
Serious/severe symptoms	4 (2.5%)	11 (7.0%)
Daily routine difficulty		
	No or mild MD	Anxiety or depression
		disorder (moderate to severe)
Daily routine difficulty GAD-7 (Anxiety)		
n	105	32
Not difficult at all	41 (39.0%)	1 (3.2%)
Somewhat difficult	54 (51.4%)	17 (54.8%)
Very difficult	9 (8.6%)	8 (25.8%)
Extremely difficult	1 (1.0%)	5 (16.1%)
Daily routine difficulty PHQ-9 (Depression)		
n	103	33
Not difficult at all	29 (28.2%)	0 (0.0%)
Somewhat difficult	62 (60.2%)	14 (42.4%)
Very difficult	11 (10.7%)	13 (39.4%)
Extremely difficult	1 (1.0%)	6 (18.2%)

Values are given as total number and %, the framing indicates the subgroup with moderate to severe MD.

Demographic data, diagnosis, functional class, hemodynamic values and medical therapy of the study population are summarized in Table 2.

**Table 2:** Study population and baseline characteristics

		**No or mild**	**Anxiety or depression**
	**All patients**	**Mental disorder**	**Disorder (moderate to severe)**
Patients, n	158	122	36
Gender male/female	45/133	39/83	6/30
Age, years	56 ± 16	57 ± 16	53 ± 16
Height, cm	167 ± 8	168 ± 8	166 ± 8
Weight, kg	75 ± 19	75 ± 17	78 ± 23
**WHO functional class-no. (%)**			
I	1 (0.6%)	5 (7.6%)	5 (8.6%)
II	16 (10.1%)	14 (11.5%)	2 (5.6%)
III	139 (88.0%)	106 (86.9%)	33 (91.7%)
IV	2 (1.3%)	1 (0.8%)	1 (2.8%)
**Diagnosis**			
Pulmonary arterial hypertension	129 (81.7%)	100 (82.0%)	29 (80.6%)
PH due to left heart disease	1 (0.6%)	1 (0.8%)	0 (0.0%)
PH due lung disease	6 (3.8%)	5 (4.1%)	1 (4.3%)
CTEPH	20 (12.7%)	15 (12.3%)	5 (13.9%)
other	2 (1.3%)	1 (0.8%)	1 (2.8%)
**Cardiac catherization:**			
Pulmonary artery pressure [mmHg]	50 ± 17	51 ± 17	49 ± 17
Pulmonary vascular resistance [dyn × sec × cm-5]	873 ± 531	865 ± 537	894 ± 520
Pulmonary capillary wedge pressure [mmHg]	9 ± 5	10 ± 6	9 ± 4
Cardiac Index [l×/in/m^2^]	2.4 ± 0.7	2.4 ± 0.6	2.4 ± 0.8
Cardiac output [l/min]	4.3 ± 1.3	4.3 ± 1.2	4.3 ± 1.4
**PAH-targeted medication**			
Endothelin receptor antagonists	106 (67.1%)	78 (63.9%)	28 (77.8%)
Phosphodiesterase-5-inhibitors	111 (70.3%)	85 (69.7%)	26 (72.2%)
Prostanoids inhaled	31 (19.6%)	24 (19.7%)	7 (19.4%)
Prostanoids intravenous	2 (1.3%)	0 (0.0%)	2 (5.6%)
Calcium channel blockers	37 (23.4%)	28 (23.0%)	9 (25.0%)
Glivec	3 (1.9%)	2 (1.6%)	1 (2.8%)
Riociguat	7 (4.4%)	6 (4.9%)	1 (2.8%)
**Combination therapy**			
Monotherapy	50 (31.8%)	42 (34.7%)	8 (22.2%)
Dual therapy	72 (45.9%)	52 (43.0%)	20 (55.6%)
Tripletherapy	30 (19.1%)	23 (19.0%)	7 (19.4%)
Quadrupletherapy	2 (1.3%)	1 (0.8%)	1 (2.8%)
**Antidepressant drug y/n**	8/150	5/117	3/33
**Oxygen therapy y/n**	79/79	64/58	15/21
**Median survival time**	2.73 ± 1.12	2.91 ± 1.06	2.17 ± 1.30

PH = pulmonary hypertension, CTEPH = chronic throboembolic pulmonary hypertension, y = yes, n = no.

### Comparison of subgroups

At baseline both groups did not significantly differ in their demographic data as age, height, weight, nor in the severity of pulmonary hypertension as WHO-functional class and hemodynamic parameters (Table 2). Patients were also comparable in their PH-targeted treatment and physical exercise capacity, measured by 6-minute walking distance and cardiopulmonary exercise testing (Table 3).

**Table 3:** Cardiopulmonary exercise testing and quality of life

		**No or mild**	**Anxiety or depression**	
**Characteristic**	**Total**	**Mental disorder**	**Disorder (moderate to severe)**	**p-value**
	**158**	**122**	**36**	
**6MWD, Meter**	**423 ± 116**	**431 ± 118**	**396 ± 106**	**0.162**
**Cardiopulmonary exercise testing**				
peak VO_2_/kg, mL/Min/kg	12.4 ± 3.6	12.5 ± 3.7	11.9 ± 3.2	0.414
peak VO_2_, ml/min	938 ± 385	946 ± 412	911 ± 275	0.652
EqCO_2_ at AT, ml/min	46.3 ± 11.1	46.8 ± 10.9	44.2 ± 11.5	0.309
VO_2_ at AT, ml/min	701 ± 222	693 ± 229	736 ± 194	0.442
Oxygen pulse, (mL/min)/min-1	7.5 ± 2.2	7.4 ± 2.3	7.6 ± 2.2	0.693
HR rest, min-1	78 ± 14	78 ± 12	77 ± 18	0.814
HR max, min-1	123 ± 20	124 ± 20	22 ± 22	0.652
RR sys rest, mmHg	115 ± 16	116 ± 17	110 ± 13	0.037
RR dia rest, mmHg	76 ± 15	77 ± 16	70 ± 9	0.019
RRsys max, mmHg	145 ± 24	148 ± 24	136 ± 24	0.010
RR dia max, mmHg	85 ± 14	86 ± 13	81 ± 15	0.090
Oxygen saturation rest, %	94 ± 5	94 ± 4	95 ± 5	0.289
Oxygen saturation max, %	88 ± 9	88 ± 9	91 ± 8	0.075
sPAP rest, mmHg	59 ± 20	59 ± 20	61 ± 20	0.628
sPAP max, mmHg	93 ± 26	92 ± 24	95 ± 30	0.589
Workload max, W	65 ± 26	65 ± 26	65 ± 23	0.990
Borg dyspnoe	15 ± 2	14 ± 3	16 ± 2	0.043
Borg PE	15 ± 2	15 ± 2	16 ± 2	0.070
**Quality of life [SF-36]**				
Physical functioning	37.5 ± 25.9	42.1 ± 26.6	21.4 ± 14.6	<0.001
Physical role performance	37.4 ± 40.9	44.3 ± 42.3	14.4 ± 25.8	<0.001
Bodily pain	70.5 ± 29.6	74.4 ± 29.3	57.2 ± 27.1	0.002
General health perceptions	40.3 ± 19.1	43.5 ± 19.3	30.4 ± 15.1	<0.001
Vitality	45.5 ± 18.6	49.5 ± 17.5	31.7 ± 15.5	<0.001
Social functioning	65.9 ± 28.8	73.9 ± 25.1	39.6 ± 24.2	<0.001
Emotional role performance	66.4 ± 48.4	76.0 ± 45.1	34.4 ± 45.9	<0.001
Mental health	65.9 ± 18.6	71.3 ± 15.3	47.9 ± 17.6	<0.001
**Laboratory parameters**				
NT-proBNP; pg/ml	1461 ± 2246	1477 ± 2413	1407 ± 1539	0.873

Values are mean ± Standard deviation; p-values are the same for absolute values differences; 6MWD, Cardiopulmonary Exercise Testing: two-sided Student t-test, Borg Scale: Wilcoxon Rank test 6-MWD = 6-minute walking distance, VO2/kg = max.oxygen consumption/kg, EqCO2 = Ventilatory equivalent for carbon dioxide, AT = anaerobic treshold HR = heart rate, RR = Blood pressure, sys = systolic, dia = diastolic, sPAP = systolic Pulmonary arterial pressure, W = Watt.

Gender distribution significantly differed between the two groups (p < 0.001), with a higher proportion of female patients in the group with moderate to severe MD (female-to-male ratio 5:1 vs. 2.1:1).

**Figure 1:**
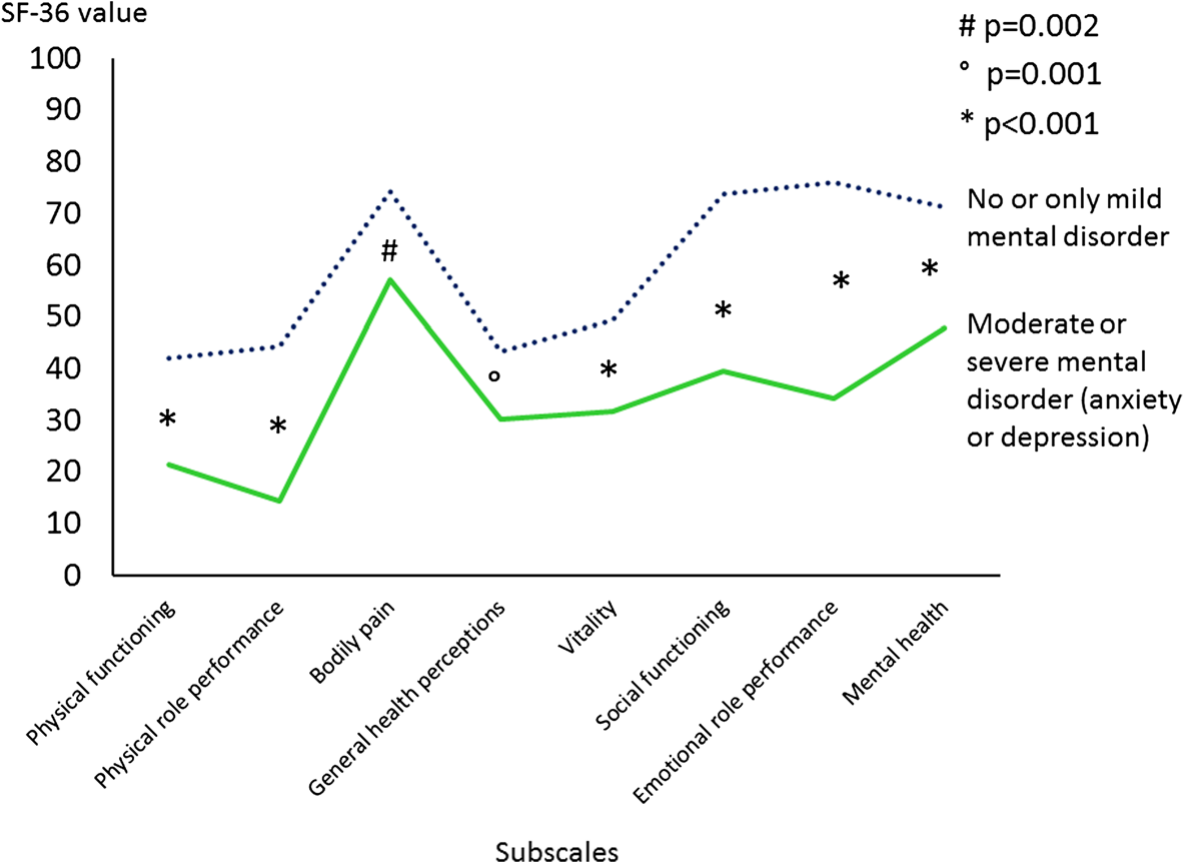
**Quality of life in patients with no or only mild MD and moderate to severe MD.** There was a significant difference between the two subgroups in relation to the SF-36 Questionnaire in all subscales (p < 0.002).

**Figure 2:**
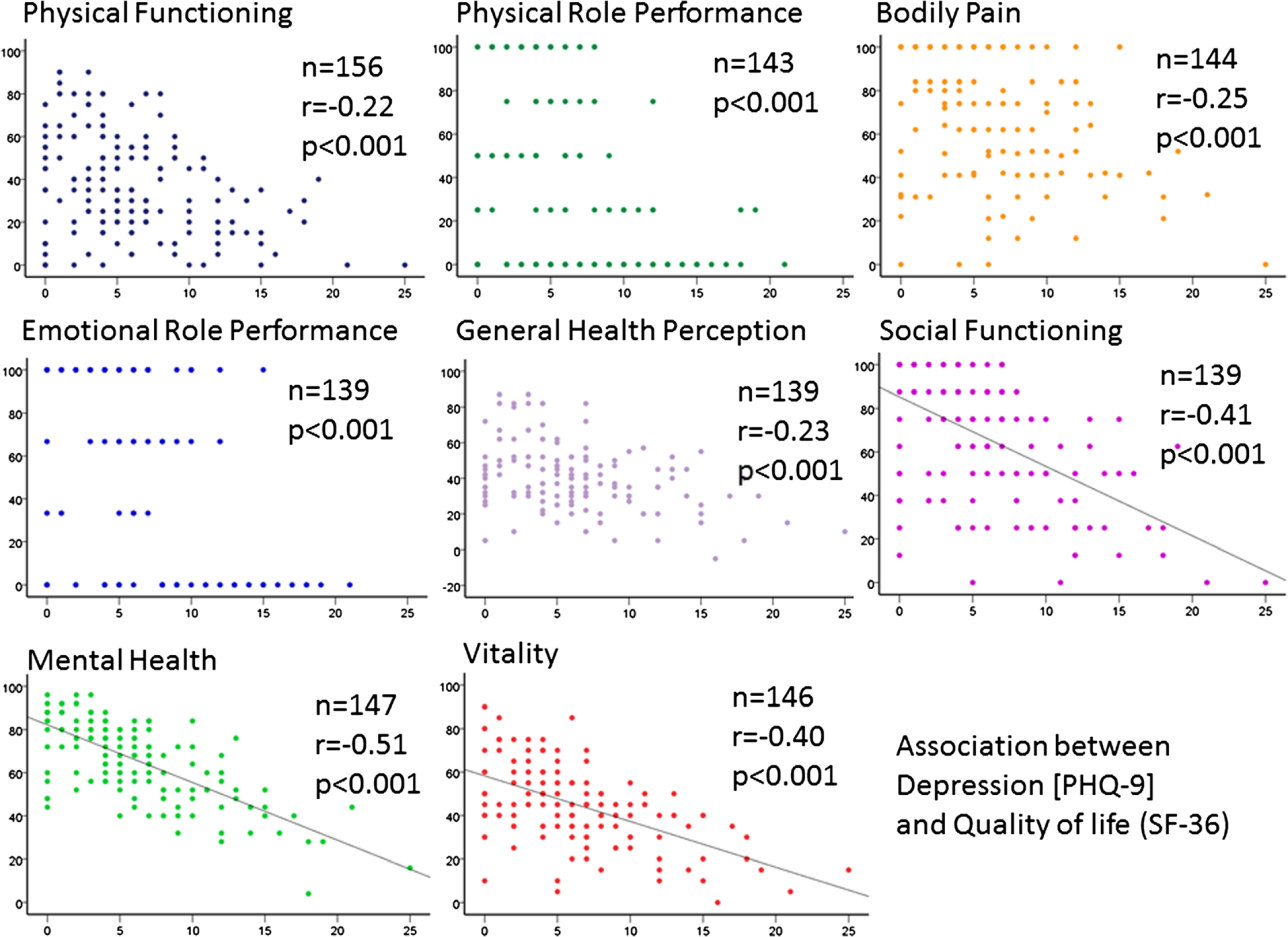
**Correlation between the subscales of quality of life (SF-36; y-axis) and depression disorder (PHQ-9, x-axis).** There was a significant correlation between the SF-36 subscales and depression score (p < 0.001 in all subscales). The highest correlation could be detected in the subscales vitality (r = −0.40) and mental health (r = −0.51). A linear regression line is only shown in the subscales social functioning, vitality and health (r-value >0.40), as only these showed evidence for a linear correlation.

Patients with moderate to severe anxiety and depression disorder (group 2) showed a significantly lower quality of life in all subscales of the SF-36 questionnaire (bodily pain p = 0.002, all other subscales p < 0.001; Figure 1, Table 3) than patients in group 1. A correlation between values of PHQ-9 (Depression Disorder) and SF-36 (Quality of Life) in all 8 subscales was detected (p < 0.001) (Figure 2). The subscales Mental health (r = −0.51) and Vitality (r = −0.40) correlated best with the severity of depression.

**Figure 3:**
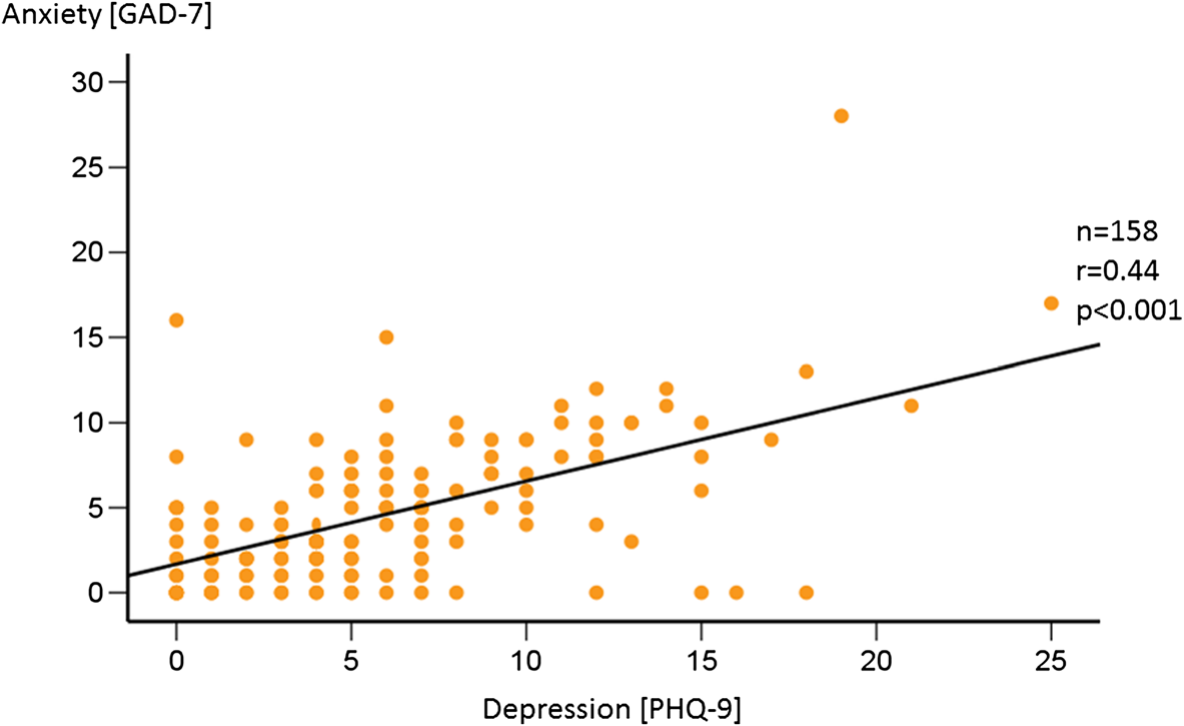
**Anxiety and depression significantly correlate in patients with PH.** Generalised Anxiety disorder (GAD-7) and Depression Disorder (PHQ-9) scales of each patient were analysed by correlation analysis. Score values significantly correlated (r = 0.44, p < 0.001) between groups.

Furthermore, there was a significant correlation between the severity of anxiety and depression disorders (Figure 3). Patients with a higher value of the GAD-7 had also higher values in PHQ-9 (r = 0.44, p < 0.001).

Significant differences were found between the magnitude of anxiety or depression disorder and the subjective daily routine difficulty. Patients with moderate-to-severe MD reported a more difficult daily routine due to anxiety with the intensity very difficult to extremely difficult in 9.6 vs. 41.9% (p < 0.001, Table 1). Impairment of daily activities due to depression was even higher with 11.7 in patients with no or mild MD vs. 57.6% in moderate to severe MD (p < 0.001).

During cardiopulmonary exercise testing, patients in subgroup 1 (without or with mild mental disorders) had significantly lower mean systolic (RRsys) and diastolic (RRdiast) systemic blood pressures at peak exercise (RRsyst rest p = 0.04, RRdiast rest p = 0.02, RRsyst max p = 0.01). Mean Borg Scale for dyspnea (p = 0.043) was significantly higher in patients without MD (Table 3). Borg Scale for peripheral exhaustion did differ between groups in trend (p = 0.07).

Nine percent of patients with moderate to severe MD received pharmacological treatment.

**Figure 4:**
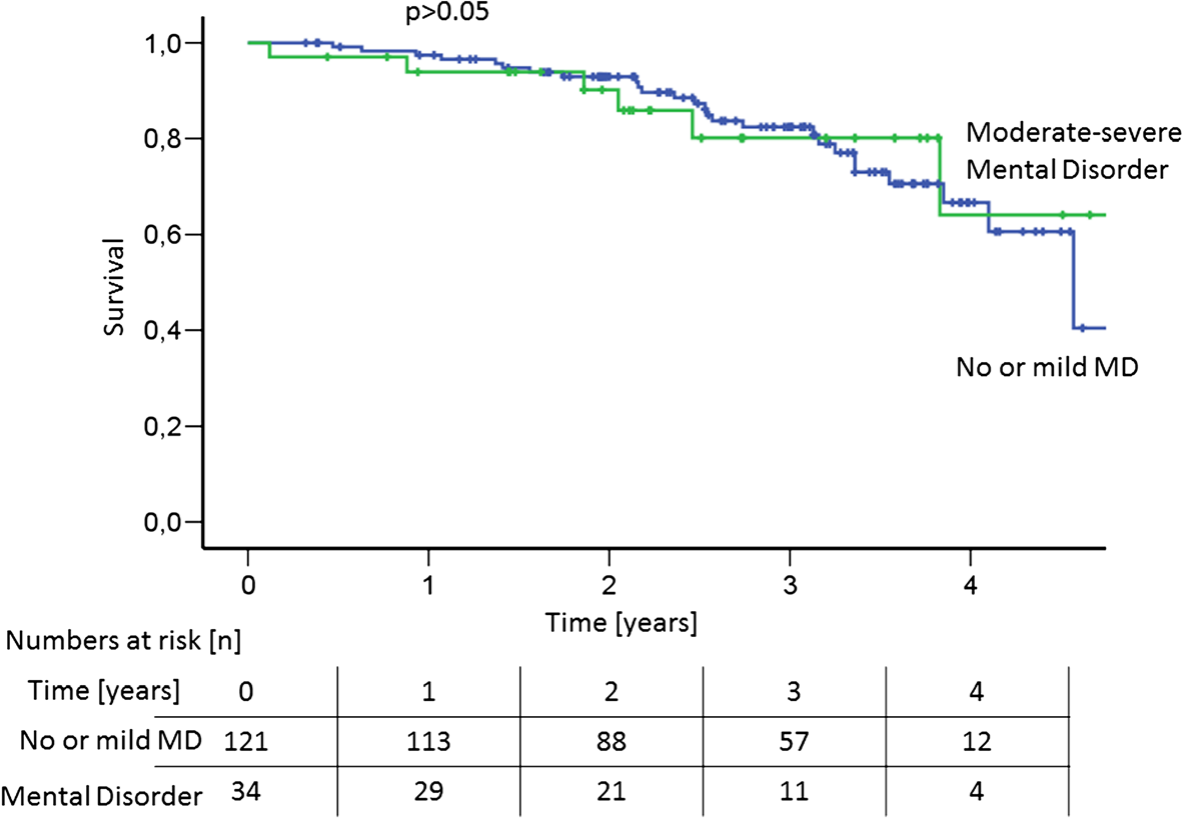
**Survival amongst PH patients according to mental disorder.** The two groups did not sigificantly differ in their survival (p > 0.05).

Overall 1-, 2- and 3- year survival was 96.7, 92.4 and 81.8%, respectively. There was no significant difference in survival between patients of group 1 with no or mild MD (1-, 2- and 3- year survival 97.4, 92.9 and 82.4%) compared to patients of group 2 with moderate to severe MD (93.9, 90.2 and 80.2%; p > 0.05) (Figure 4).

## Discussion

The results of this prospective study show for the first time that mental disorders as anxiety and depression are significantly associated with an impaired quality of life in PH-patients but may not be associated with reduced survival. The study confirms the previously reported high prevalence of mental disorders in these patients. In only a small proportion of patients MD have been medically treated.

### Correlation of depression and anxiety disorders with quality of life

Patients with moderate to severe mental disorder (group 2) had a significantly lower quality of life shown in all subscales of SF-36. Despite promising new medical treatment options, QoL still constitutes one of the main treatment goals of PH therapy [21]. By proving a strong association of MD with QoL in PH patients, our study highlights the importance of taking MD into account in clinical management of PH patients. Previously the association of MD with QoL has been proven in several other diseases, as in cystic fibrosis [22], diabetes mellitus [23], heart diseases [24] or even in community dwelling adults [25]. Several authors come to the conclusion to treat MD in order to improve QoL [22-25]. For example, in patients with left heart failure, remission of depression led to an increase in quality of life, 6-minute walking distance and social function [26]. In our patients, the occurrence of MD was associated with a higher perceived difficulty of performing daily activities. However it is not clear, whether this is a result of MD or a possible reason for the development of MD.

Corresponding to this finding, our data showed a higher perceived exertion in moderate to severe MD measured by Borg Scale, though maximal workload during ergometer test did not significantly differ between groups. As both subgroups did not differ in their physical exercise capacity this might be a hint for a different perception of the patients’ abilities and limitations independent of the patients’ exercise capacity. This phenomenon has to be further investigated.

The association between physical exercise capacity and quality of life has recently been shown for PAH and CTEPH [27].

### High prevalence of MD in PAH/CTEPH

This prospective study confirms the previously reported high prevalence of mental disorders in PAH [6,9,28-30] and shows a similar prevalence for inoperable CTEPH patients. The rate of moderate to severe major depressive disorder in PAH has been estimated to be 20%-50% [9,29,31] and was similar to the prevalence in patients with left-heart failure (30% major depression) [32]. In our study a female predominance was detected among PAH patients with depression or anxiety disorder (male 13.3 vs. female 26.5%) corresponding to a previously described higher likelihood of women being diagnosed with MD [33,34]. Due to the severity of the disease, pulmonary hypertension is thought to produce emotional responses of anxiety, depression and panic attacks [35].

### Mental disorders and treatment in PH

Only a few studies have assessed the effects of treatment of depression or anxiety in patients with pulmonary hypertension. Patients treated with epoprostenol had a lower rate of depression and anxiety compared to patients whithout this treatment [29]. This finding suggests that improvement of PAH-driven symptoms by PAH-targeted medical therapy or by a specialized training program [18] may improve the comorbidity with anxiety and depression. However, clinical significance and optimal therapy strategy of MD in patients with PH are unclear. In patients with left heart failure, remission of depression led to an increase in quality of life, 6-minute walking distance and social function [26]. A study investigating the effect of antidepressant medication on morbidity and mortality in depressed patients after myocardial infarction showed a slightly reduced cardiovascular mortality [36]. In our study, the clinicians who treated the patients were blinded to the results of the MD-targeted questionnaires. Therefore, in many patients of the study the participating clinicians may have overseen the MDs. In fact 91% of our patients with moderate-to-severe MD were not receiving psychopharmacological treatment. Similar observations have previously been made with 75% of PH-patients with mild-to-severe depression not receiving antidepressant therapy [9]. Taking into account that the manifestation of MD was highly correlated with a reduced quality of life, we can conclude that there is still a lack of psychosocial support for PH patients. However, the impact of counseling and integration of psychosocial support and pharmacological treatment of patients with PH have to be further investigated by randomized controlled trials. In addition, long term data have to be assessed in order to investigate the cause of MD, as it is not clear, whether a decrease in QoL or MD develops first during the clinical course of the disease. Attention to MD in any patient population seems to be useful, but it’s not clear that an association suggests causality, and in fact it may well be that it is the QoL limitations which produce anxiety and depression.

From our clinical experience exercise training as add-on to medical therapy, which has been effective to improve work capacity, quality of life and further prognostic relevant parameters in patients with PH [18,37-40], may also be helpful to improve depression and anxiety. In patients with coronary artery disease exercise training improved depressive symptoms and survival [41]. Therefore, it might also be worth to investigate the effect of this add-on treatment on MD in PH.

### Mental disorders in PH and survival

Despite no specific treatment of MD in the majority of patients, our study did not detect a significant difference in survival between patients with no or mild MD compared to patients with moderate to severe MD. So far, it is still uncertain whether depressive episodes in patients with PH, as in reactive depression, are mechanisms belonging to the phases of coping. However, in this study we did not obtain follow-up data of the patients’ clinical and mental course. It is possible that mental disorders obtained within the study have improved during follow-up or may have been treated at a later stage. Therefore, the data of this study are not conclusive regarding the association of MD with survival or time to clinical worsening.

## Conclusion

Anxiety and depression were frequently diagnosed in patients with PAH and inoperable CTEPH despite optimized medical PH-treatment and significantly correlated with a reduced quality of life. Most patients did not receive psychopharmacological treatment. MD in PH appears to be not only underdiagnosed but also undertreated. The results of this study suggest that a screening for MD in PH-patients using specific questionnaires should be integrated in clinical practice. Further randomized controlled studies are needed to assess the effect of specific MD-targeted treatment on quality of life and survival in PH-patients.

## Competing interest

The authors declare that they have no competing interest.

## Authors’ contributions

EG, HK, DH, NE, MH made substantial contributions to conception and design. EG, CN, NE, FP acquired the data. CF and NE analysed and interpreted the data; DH, HK, FP, NE, CN, CF, AG, SR, HJS, MH, EM, EG and SG participated in drafting the article and revised it critically for important intellectual content. All authors read and approved the final manuscript.
